# Comparative Performance of Machine Learning Models and Logistic Regression for Acute Myocardial Infarction Risk Prediction in the COVID-19 Vaccination Era: A Retrospective Observational Study

**DOI:** 10.7759/cureus.107396

**Published:** 2026-04-20

**Authors:** Vasileios Georgakis, Panos Xenos

**Affiliations:** 1 Department of Statistics and Insurance Science, University of Piraeus, Piraeus, GRC

**Keywords:** actuarial modeling, covid-19 vaccination, healthcare administration, machine learning, myocardial infarction, risk stratification

## Abstract

Introduction

The integration of Artificial Intelligence in healthcare systems offers transformative potential for enhancing healthcare administration and improving insurance risk modeling through more accurate patient stratification. This study evaluates the predictive performance of support vector machines (SVM), gradient boosting (GBM), and random forest (RF) against multivariate logistic regression for Acute Myocardial Infarction (AMI) risk stratification, focusing on its implications for healthcare administration and insurance modeling.

Methods

A retrospective observational study was conducted on 901 patients at Alexandra Hospital of Athens (January 2021 to December 2023). Inclusion was restricted to first-incident AMI cases, verified through clinical and digital registries; patients with a prior history of AMI were strictly excluded. Predictor variables included standardized clinical data, lifestyle factors, and COVID-19 vaccination status, all verified prior to the AMI event. Models were trained and validated using a 70/30 hold-out split (n=631 training; n=270 testing), a strategy selected to provide a stable testing set for benchmark comparison. Performance was evaluated based on accuracy, sensitivity, and area under the curve (AUC) to assess how predictive precision can enhance organizational risk assessment and resource allocation.

Results

Among the predictive models, SVM achieved the highest overall accuracy (62.08%), while GBM demonstrated a modest discriminative capacity with an AUC of 0.63 and a sensitivity of 0.76. Crucially, within this specific clinical cohort, COVID-19 vaccination status showed no statistically significant association with AMI risk (OR 1.12, p=0.41). These results represent an exploratory comparison of algorithmic performance for risk prediction rather than a finalized tool for robust clinical stratification.

Conclusion

While the accuracy gain over logistic regression is incremental, SVM and GBM models provide a more refined classification for high-risk cohorts. These findings suggest that AI-based stratification can optimize risk management frameworks and actuarial modeling, effectively bridging clinical cardiology with insurance science.

## Introduction

Cardiovascular diseases (CVDs) continue to be the leading cause of mortality worldwide, placing an immense burden on healthcare systems. Among them, acute myocardial infarction (AMI) requires rapid and precise risk stratification to enhance healthcare administration and optimize resource allocation in intensive care units (ICUs) [1]. In the post-pandemic era, identifying modifiable risk factors is crucial for updating prevention guidelines and improving insurance risk modeling [2].

In recent years, there has been a surge in enthusiasm for machine learning (ML) algorithms, such as support vector machines (SVMs) and gradient boosting machines (GBMs), which are often compared to traditional generalized linear models to evaluate their predictive superiority [3,4]. However, while these advanced models excel in high-dimensional tasks, their practical advantage in handling structured, tabular clinical data for hospital management is not always clear-cut. Furthermore, the global COVID-19 vaccination campaign has introduced a new variable into clinical risk assessment. Providing real-world evidence on the association between vaccination status and AMI has become a public health priority to address clinical concerns and inform patient management.

The objectives of this study were to evaluate classic and emerging risk factors for AMI in a cohort of 901 patients, to investigate the potential association between AMI and COVID-19 vaccination status, and to compare the efficacy of advanced ML algorithms (SVM, GBM, RF) against standard logistic regression in a real-world clinical setting.

## Materials and methods

This retrospective observational study was conducted at a tertiary university hospital in Athens, Greece (Alexandra General Hospital). Formal ethical approval for the research protocol and data collection was granted by the hospital’s Scientific Council on September 28, 2022 (Protocol No. 84). Following this institutional approval, data extraction and clinical review were performed for the period between November 2022 and April 2023. Inclusion was strictly limited to first-incident acute myocardial infarction (AMI) cases. To ensure the integrity of the risk prediction model and focus on incidence, patients with a documented prior history of AMI were strictly excluded.

Variables

The primary outcome of this study was the clinical diagnosis of first-incident AMI. Independent variables were selected based on the internationally established risk factors identified in the INTERHEART study [5], which demonstrated that nine modifiable factors account for the vast majority of AMI risk globally. To ensure the temporal precedence required for a predictive model, all exposure variables, including lifestyle factors, clinical history, and vaccination status, were verified as having occurred prior to the onset of the AMI event through a review of hospital electronic records and clinical history-taking at the time of admission.

Specifically, smoking was defined as current daily use or cessation within the past year. Family history was strictly defined as a positive history of premature coronary artery disease in a first-degree relative (males <55 years, females <65 years). Hypertension and diabetes mellitus were identified based on documented clinical diagnoses or the chronic use of anti-hypertensive and anti-diabetic medications, respectively. Systemic allergy was recorded as a binary variable representing a history of allergic reactions to drugs, food, or environmental factors. Furthermore, a clear distinction was made between chronic cardiovascular treatments (e.g., statins) and illicit drug use (e.g., cocaine or amphetamines), which were evaluated as separate risk factors due to their known association with acute cardiac events. Due to the rigorous manual data verification process and strict inclusion criteria, a 0% missing value rate was achieved for all primary predictors in the final cohort of 901 patients.

Statistical analysis

Data analysis was conducted using the R programming environment (v4.3.3) with the caret, e1071, gbm, and pROC packages. To ensure data uniformity and model convergence, all continuous variables were standardized using Z-score normalization. Given that the AMI incidence was 45.1%, the dataset was considered naturally balanced, and no further resampling (e.g., SMOTE) was required.

The cohort was stratified and split into a training set (70%, n=631) and an independent test set (30%, n=270). To optimize the machine learning algorithms and prevent overfitting, we employed a 10-fold cross-validation strategy during the training phase. For predictive modeling, specific hyperparameters were selected to balance accuracy and interpretability; the SVM was implemented with a linear kernel, while the GBM utilized a Bernoulli distribution with 500 trees and a shrinkage factor of 0.01.

Baseline associations were estimated via multivariable logistic regression to provide odds ratios (OR) and 95% confidence intervals (CI). Variables were screened using univariable logistic regression, and those with a p-value < 0.20 were considered for inclusion in the final multivariable model, which was then developed using a backward stepwise elimination procedure until a parsimonious model was achieved.

The predictive performance of SVM and GBM was compared on the unseen test data using accuracy, sensitivity, and area under the curve (AUC). Classification metrics (accuracy, sensitivity) were derived from 2x2 confusion matrices using a fixed probability cutoff score of 0.5. All tests were two-tailed, with statistical significance set at p < 0.05.

## Results

The study population included 901 individuals, with an AMI prevalence of 45.1% (n=406). The multivariable logistic regression analysis (Table 1) revealed that smoking remains the most potent predictor of infarction in our cohort (odds ratio (OR): 1.89; 95% confidence interval (CI): 1.41-2.53; P < 0.001). Male sex was also significantly associated with increased odds of AMI (OR: 1.46; 95% CI: 1.09-1.96; P = 0.01). For all statistical tests, a p-value of < 0.05 was considered the threshold for statistical significance.

**Table 1:** Multivariable logistic regression analysis of risk factors for acute myocardial infarction (n = 901) Data are presented as absolute counts (n) and percentages (%) for categorical variables, or mean ± standard deviation (SD) for continuous variables. Results are expressed as crude (univariable) and adjusted (multivariable) odds ratios (ORs) with a 95% confidence interval (CI). Variables with a univariable p-value < 0.20 were considered for inclusion in the final multivariable model, which was then refined using a backward stepwise elimination procedure (p-value threshold < 0.05). A p-value of < 0.05 was considered statistically significant. SD: Standard Deviation; OR: Odds Ratio; CI: Confidence Interval

Predictors (Risk Factors)	Crude OR (95% CI)	Adjusted OR (95% CI)	P-value (Crude)
Smoking (Yes vs No)	1.85 (1.42 – 2.41)	1.89 (1.41 – 2.53)	< 0.001
Gender (Male vs Female)	1.59 (1.20 – 2.12)	1.46 (1.09 – 1.96)	0.001
Age (High vs Low)	0.77 (0.59 – 1.00)	—	0.049
Illicit Drugs (Yes vs No)	0.46 (0.22 – 0.88)	—	0.023
Vaccination Status	1.13 (0.87 – 1.47)	1.12 (0.85 – 1.48)	0.371
Cardiovascular Meds	0.86 (0.66 – 1.12)	0.28 (0.12 – 0.65)	0.256
Allergies	0.75 (0.51 – 1.10)	—	0.148
Blood Pressure (BP)	1.09 (0.83 – 1.43)	—	0.535
Glucose (Diabetes)	1.15 (0.87 – 1.51)	—	0.327
Alcohol Use	0.93 (0.59 – 1.46)	—	0.765
Psychological Stress	0.95 (0.66 – 1.36)	—	0.792
Family History	0.98 (0.75 – 1.28)	—	0.904

**Figure 1:**
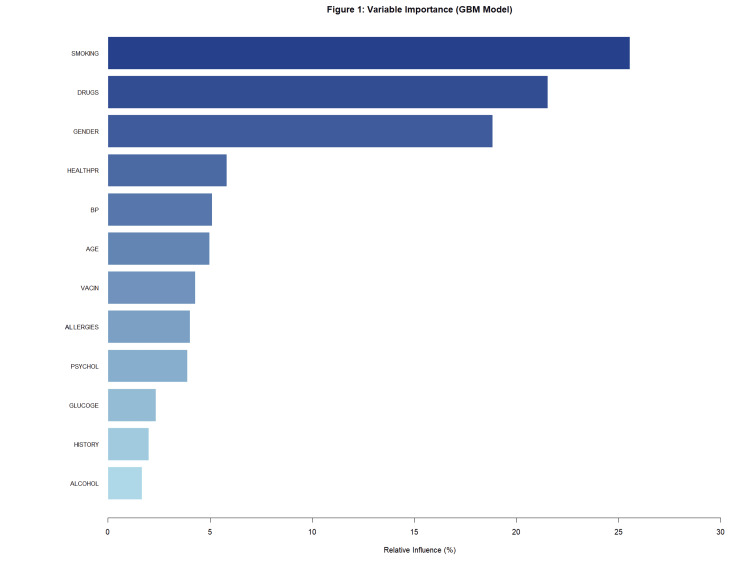
Relative importance of predictors in the final machine learning model Feature importance plot ranking the clinical and demographic variables used to predict acute myocardial infarction (AMI). The rankings are based on the relative influence (%) calculated by the gradient boosting machine (GBM) algorithm. Age, smoking status, and blood pressure levels were identified as key predictors, whereas COVID-19 vaccination status (VACIN) showed minimal influence on model outcomes.

The relative importance of each clinical and lifestyle variable was further analyzed using the GBM algorithm. As illustrated in Figure 1, smoking and male gender were identified as the most influential predictors, followed by age and blood pressure. Notably, vaccination status (VACIN) showed a significantly lower relative influence compared to traditional risk factors, reinforcing its non-significant role in AMI occurrence in this cohort. For all statistical analyses, a p-value of <0.05 was considered the threshold for statistical significance.

Multivariable logistic regression analysis revealed that smoking remains the most potent predictor of infarction in our cohort (OR: 1.89; 95% CI: 1.41-2.53; P < 0.001). Male sex was also significantly associated with increased odds of AMI (OR: 1.46; 95% CI: 1.09-1.96; P = 0.01). Furthermore, multivariable logistic regression analysis was performed to evaluate the role of COVID-19 vaccination as a predictor for AMI, adjusting for the significant predictors identified in the final model (smoking status and gender). The analysis revealed no statistically significant association between vaccination status and AMI incidence (OR: 1.12; 95% CI: 0.85-1.48; P = 0.41). As illustrated in the variable importance plot (Figure 1), VACIN demonstrated a negligible relative influence on the model’s overall predictive performance. For all statistical tests, a p-value of < 0.05 was considered the threshold for statistical significance. 

Comparative model performance

We evaluated the predictive performance of the models on the unseen test data (Table 2). The SVM achieved the highest overall accuracy of 62.08%. In terms of discriminative capacity, the GBM yielded a nominally higher AUC (0.63) compared to logistic regression (AUC: 0.62) and SVM (AUC: 0.60). However, statistical comparison using DeLong’s test indicated that these differences were not significant (GBM vs. logistic regression: p=0.74; GBM vs. SVM: p=0.48), suggesting comparable discriminative performance across the models. Despite this statistical parity in AUC, GBM demonstrated the highest sensitivity (0.76), followed by logistic regression (0.74). These results suggest that while the models are overall comparable, ensemble methods like GBM may offer a slight advantage in clinical risk stratification by prioritizing the minimization of false negatives (Type II errors).

**Table 2:** Performance metrics and comparative analysis of the predictive models on the test set Evaluation of model efficacy using an independent validation subset (30% of the total cohort). The training phase incorporated a 70/30 stratified split with cross-validation to ensure internal consistency. Accuracy is presented as a percentage (%), while sensitivity and AUC are reported as decimal values. A p-value < 0.05 was considered the threshold for statistical significance. GBM: Gradient Boosting Machine; Log. Reg.: Logistic Regression; AUC: Area Under the Curve (ROC); Sensitivity: True Positive Rate

Model	Accuracy (%)	Sensitivity	AUC	Performance Assessment
SVM (Linear)	62.08%	0.71	0.60	Highest Accuracy
GBM	60.22%	0.76	0.63	Best Discriminator (AUC/Sens)
Log. Reg.	59.48%	0.74	0.62	Balanced & Interpretable

**Figure 2:**
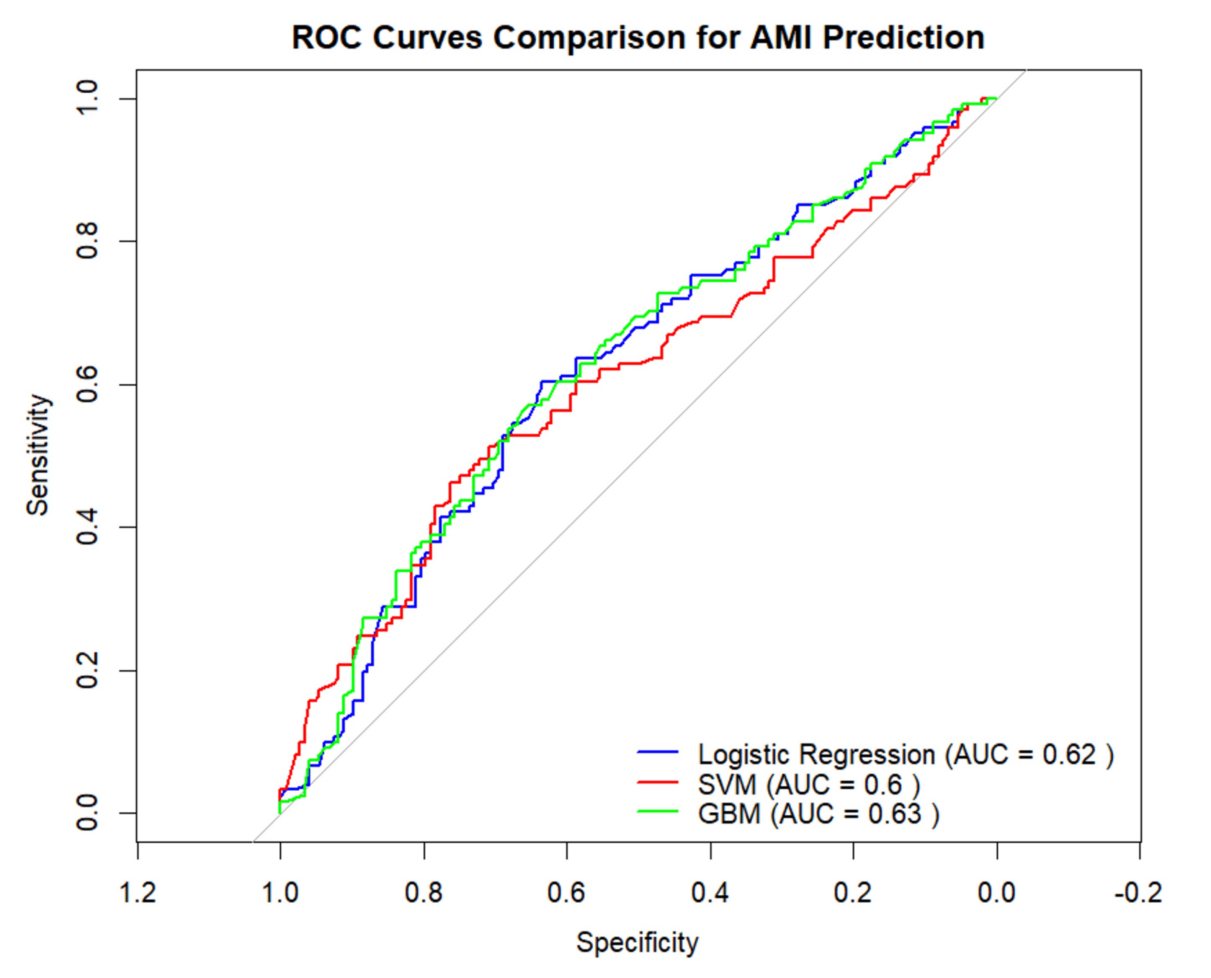
Comparative ROC curves for predictive performance assessment Receiver operating characteristic (ROC) curves evaluating the performance of the gradient boosting machine (GBM), logistic regression, and support vector machine (SVM) models. The area under the curve (AUC) values demonstrate that the GBM model (AUC = 0.63) achieved the highest discriminative capacity for predicting acute myocardial infarction (AMI).

The discriminative capacity of the models was further validated through receiver operating characteristic (ROC) analysis (Figure 2). Among the tested algorithms, the GBM model (green curve) yielded the highest AUC (0.63). While this AUC value is considered modest by traditional diagnostic standards, and pairwise comparisons using DeLong’s test indicated that the differences between models were not statistically significant (p > 0.05), the GBM demonstrated a nominally higher discriminative potential. Critically, despite this modest aggregate discrimination, the GBM’s high sensitivity (0.76) suggests its practical utility as an exploratory benchmark for AMI screening in real-world settings characterized by the inherent noise of retrospective, non-digitized hospital data.

## Discussion

Our study highlights that traditional risk factors, specifically smoking and male sex, remain the primary determinants of myocardial infarction. The significance of comorbidities and lifestyle factors in predicting incident myocardial infarction has been extensively documented in recent algorithmic studies [6], which underscore the importance of personal health history in clinical risk assessment. Furthermore, machine learning approaches have demonstrated clinical value in predicting long-term cardiac death and infarction risk based on combined clinical markers [7]. The manual verification of our data adds weight to these findings, suggesting that lifestyle modifications should remain the cornerstone of preventive cardiology.

The "complexity trap" and organizational value

One of the most significant takeaways from this research is the comparison between simple and complex models. While sophisticated algorithms like GBM are theoretically powerful, our results show that their advantage in structured clinical datasets depends on the evaluation metric used. Although SVM achieved the highest overall accuracy (62.08%), the GBM yielded a nominally higher AUC (0.63) compared to logistic regression (AUC: 0.62) and SVM (AUC: 0.60). However, formal pairwise comparisons using DeLong’s test indicated that these differences in AUC were not statistically significant (p=0.74 for GBM vs. LR; p=0.48 for GBM vs. SVM), suggesting that the models demonstrate comparable discriminative capacity in this specific cohort. This observation is consistent with the broader literature, where the trade-off between model complexity and interpretability remains a central challenge [8].

From a healthcare management and insurance perspective, these findings are critical. While machine learning has shown high efficacy in specific tasks like mortality prediction for heart failure [9] or extubation failure in ICUs [10], our study demonstrates its complementary value in actuarial modeling. Utilizing risk stratification algorithms like GBM and SVM offers a robust alternative approach for premium calculations and efficient resource allocation, providing comparable predictive performance to traditional methods while offering deeper insights into feature importance, as highlighted in contemporary predictive mortality frameworks [11].

Furthermore, a pivotal finding of our analysis concerns the safety of COVID-19 vaccination. After adjusting for potential confounders, vaccination status showed no statistically significant association with AMI (OR: 1.12; 95% CI: 0.85-1.48; p=0.41). By demonstrating a neutral association, our study provides essential evidence for public health administration, helping to mitigate vaccine hesitancy through data-driven assurance [12].

Study limitations

Despite the robust sample size of 901 patients, this study has certain limitations. First, the discriminative performance of the models (peak AUC: 0.63) did not reach the traditional 'good' threshold of 0.70, reflecting the inherent 'noise' of our retrospective dataset. Notably, the absence of dynamic, time-series clinical markers, such as high-sensitivity troponin kinetics or continuous electrocardiographic monitoring, likely limited the models' ability to capture the evolving nature of acute myocardial ischemia [11]. Second, its retrospective nature limits the ability to establish direct temporal causality. Third, as a single-center study, the findings may reflect local clinical patterns and might not be fully generalizable.

Furthermore, it is important to emphasize that due to the lack of integrated electronic health records (EHR) in the Greek public hospital infrastructure, data collection was a massive, manual undertaking. Each of the 901 records was retrieved and verified "by hand" from physical archives and fragmented digital laboratory entries. While this process is labor-intensive, this meticulous manual vetting allowed for a highly objective, case-by-case validation of each data point--a level of oversight that automated extractions often lack. This rigorous manual validation underscores our commitment to data integrity and ensures the internal validity of our predictive models despite the structural challenges of the healthcare environment.

Finally, our analysis regarding COVID-19 vaccination is subject to potential residual confounding. We lacked granular data on specific vaccine brands, the number of booster doses administered, and the prevalent SARS-CoV-2 variants during the study period. Furthermore, very few clinical parameters were available to serve as confounders for the vaccination status. Consequently, while our results showed no significant association with AMI, they should be interpreted as preliminary observations within the context of our specific model rather than a comprehensive safety evaluation. Lastly, the implementation of 10-fold cross-validation and strict exclusion of missing values was employed to further safeguard the robustness of our results.

## Conclusions

In conclusion, our analysis confirms that smoking and male sex remain the dominant predictors of AMI, while COVID-19 vaccination poses no observable cardiovascular risk. Methodologically, the superior discriminative capacity of the GBM model and the high accuracy of the SVM offer a predictive edge that can be leveraged by health organizations for advanced risk assessment and resource optimization. Despite the modest overall AUC, the consistently high sensitivity observed across all predictive models (ranging from 0.72 to 0.76) ensures that fewer high-risk patients are missed, thereby prioritizing clinical safety and supporting cost-effective screening. This level of performance across different algorithms allows for a more comprehensive actuarial risk stratification, enabling insurance providers to design data-driven health policies and effectively bridge the gap between clinical cardiology and insurance science.

Our study contributes to the clinical evidence regarding the safety profile of COVID-19 vaccines in relation to AMI. The absence of a statistically significant association between vaccination status and AMI (p = 0.41) within our cohort aligns with broader safety reports. While these observations are encouraging, they should be interpreted with caution due to the lack of specific data on vaccine types, booster doses, and viral variants. These findings support the inclusion of vaccination status in predictive models without it being a primary driver of ischemic risk in our sample.
